# How Cysteine Protease Gene *PtCP5* Affects Seed Germination by Mobilizing Storage Proteins in *Populus trichocarpa*

**DOI:** 10.3390/ijms222312637

**Published:** 2021-11-23

**Authors:** Xiatong Liu, Lijie Mo, Xiaorui Guo, Qiang Zhang, Hui Li, Di Liu, Hai Lu

**Affiliations:** 1 National Engineering Laboratory for Tree Breeding, College of Biological Sciences and Biotechnology, Beijing Forestry University, Beijing 100083, China; summertong0621@sina.com (X.L.); lijiemo18510990301@163.com (L.M.); guoxiaorui0404@163.com (X.G.); qiangzhang@scisoon.cn (Q.Z.); 830lihui@163.com (H.L.); 2 The Tree and Ornamental Plant Breeding and Biotechnology Laboratory of National Forestry and Grassland Administration, College of Biological Sciences and Biotechnology, Beijing Forestry University, Beijing 100083, China

**Keywords:** cysteine protease PtCP5, promoter, tissue-specific expression, pollen, seed germination

## Abstract

In higher plants, seed storage proteins are deposited in protein storage vacuoles (PSVs) and degraded by protease, especially cysteine proteases, as a source of nitrogen for seed germination. In this study, a cathepsin B-like cysteine protease PtCP5, which is important for seed germination and pollen development, was first cloned in Populus trichocarpa. The GUS staining of the Pro*PtCP5*-GUS reporter line showed that PtCP5 is expressed in the roots, stems, leaves, flowers, siliques and seeds of Arabidopsis. We reveal that PtCP5 is present in plasma membrane and co-localizes with the plasma membrane marker REM1.3. Both seed germination and early seedling development are slower in OX-PtCP5 transgenic Arabidopsis when compared with the wild-type. Further analysis revealed that, when stained with toluidine blue, the observed storage protein accumulation was lower in OX-PtCP5 than in the wild-type. Our results also show that the number of abnormal pollen grains is higher and the germination rate of pollen is lower in OX-PtCP5 than in the wild-type. These results indicate that PtCP5 is an important factor in mobilizing storage proteins and that the proper expression of PtCP5 is necessary for both pollen and seed maturation and germination. This study sheds further light on the biological functions of cysteine proteases and provides further reference for seed development research on woody plants.

## 1. Introduction

Plant cysteine proteases are widely involved in protein maturation, reconstruction and degradation during plant growth and development [1]. When grain seeds germinate, protease is released from scutellum epithelial cells and the aleurone layer into the endosperm, which then degrades the storage protein [2]. The amino acids produced, along with small peptides, are absorbed by the scutellum, and then transported to growing seedlings, providing the necessary amino acids for the growth and development of seedlings [3,4]. The normal activation of cysteine protease is key to ensuring regular seed germination as well as seedling growth and development [5,6]. Therefore, it is necessary to strictly control the start-up time node of protease activity to avoid the degradation of protein during the nongermination period in order to encourage seed germination.

Gliadin is the main storage protein in cereal seeds. Cysteine protease can degrade about 90% of gliadin during the germination of maize and wheat seeds. In barley, 42 proteases are involved in seed germination, 27 of which are cysteine proteases [7]. Papain-like cysteine proteases (C1A) and vacuolar processing enzymes (VPE), which are responsible for degrading seed storage proteins and providing nutrition for seedling growth, are the main proteases involved in the seed germination of dicotyledons and monocotyledons [1,2,5]. Previously, research has reported that the seed-type βVPE is the most important for the maturation of storage proteins in *Arabidopsis* seeds [8]. *Arabidopsis* δVPE is expressed in layers ii2 and ii3 of the seed’s coat during early seed development and is involved in the cell death of specific layers during seed coat development [9]. Both EPA and EPB degrade gliadin. EP8, an EPA-homolog cysteine proteases found in black wheat, which is responsible for mobilizing stored proteins during seed germination, is synthesized in the aleurone layer during seed germination and is inhibited by endogenous cystatin TRCC-4 [3]. EPB is expressed in epithelial cells after seed germination and is then localized to the aleurone tissue around the endosperm [10]. HvPaP-4, HvPaP-6 and HvPaP-10 are highly seed specific and are expressed in germinated seeds of barley [11]. HvPaP-1 is involved in protein mobilization during the germination of barley seeds, and the overexpression of HvPaP-1 reduces the amount of starch in seeds and increases the germination rate [12,13]. Cysteine proteases have been found to be involved in tapetal PCD during pollen development in many plants. In the cases of NtCP56 in tobacco [14], *Arabidopsis thaliana* cysteine proteases 51 (CP51), AtCP56 [15], CEP1 [16], βVPE [17], Brassica napus BnaC.CP20.1 [18] and rice OsCP1 [19], the abnormal expression of these protease genes affects the tapetal PCD process, leading to different degrees of pollen abortion.

C1A cysteine proteases can be further divided into nine subclasses according to their functional and structural characteristics, namely RD21A-like, RD19A-like, CEP1-like, XCP2-like, XBCP3-like, THI1-like, SAG12-like, AALP-like and CTB3-like cysteine proteases [20]. The primary structure of CTB3-like cysteine proteases is highly similar to that of human cathepsin B (EC 3.4.22.1; CysProt). In *Arabidopsis thaliana*, cathepsin B-like proteases are encoded by a gene family of three members: CTB1, CTB2 and CTB3. In addition, there are four additional disulfide bonds and a conserved glycosylation site to stabilize the a-helix domain, as well as a lack of corresponding floral structural elements in its structural domain [20]. *AtCathB1(AtCB1)* is almost undetectable in roots, leaves, stems, flowers and silique, while *AtCathB2(AtCB2)* and *AtCathB3(AtCB3)* appear in roots, leaves, stems, flowers and throughout silique development. The use of GUS histochemical staining during seed germination meant that the expression of the *AtCathB3* gene was detected before radicle protrusion, in the hypocotyl–radicle transition zone of the embryonic axis (12–24 h) and later (36–60 h) in the incipient vascular elements of the radicle and the hypocotyl, as well as in the cotyledons [21]. Previous reports have also shown that CTB3-like genes may regulate ER-stress-induced PCD [22] and are required for PCD development during the HR but not R gene-mediated resistance [23]. So far, there have been few studies on the physiological significance of CTB3-like genes during seed germination.

In this paper, PtCP5, a homolog gene of *Arabidopsis* AtCTB3, was cloned from *Populus trichocarpa*, and both its physiological significance and expression pattern were examined during seed germination and post-germination. Our results show that PtCP5 is specifically expressed in seeds and pollen and may participate in protein maturation to affect seed and pollen germination. The lack of PtCP5 leads to slow protein degradation, and because of this, the protein necessary for seed and pollen development cannot be supplied in time, resulting in abnormal seed and pollen germination.

## 2. Results

### 2.1. Cloning and Analysis of the PtCP5 Gene

**Figure 1:**
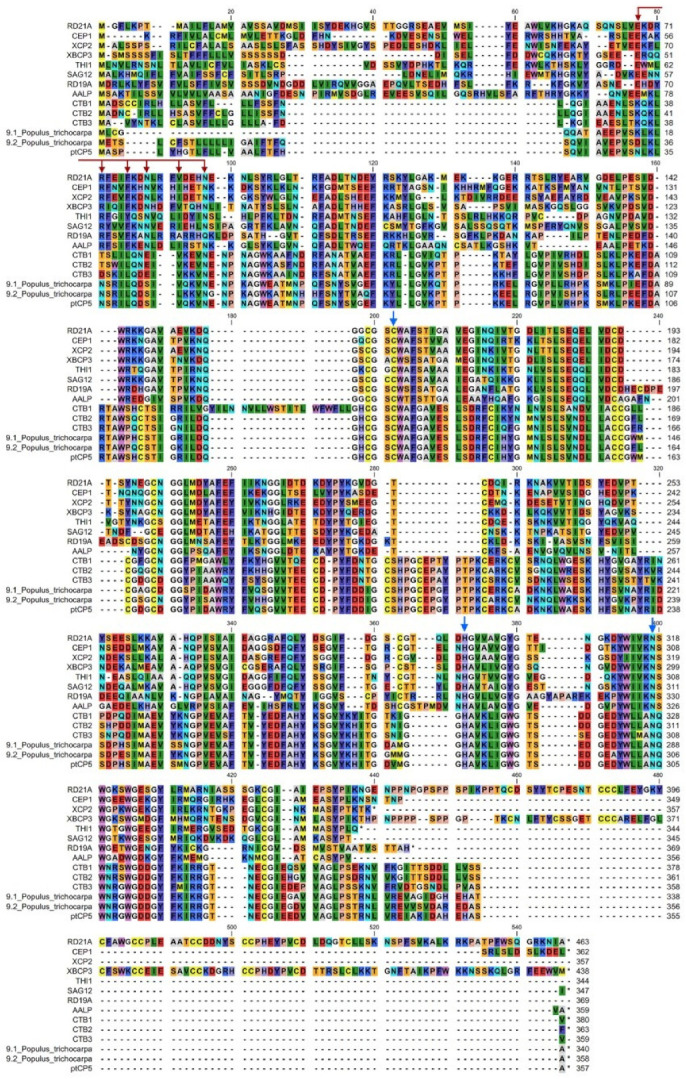
PtCP5 sequence analysis.

**Figure 2:**
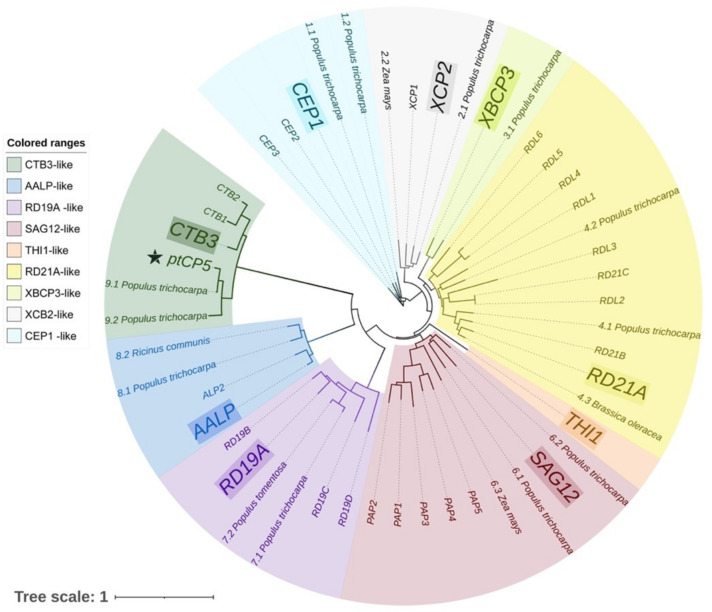
Phylogenetic analysis of PtCP5 based on an NJ tree with 1000 bootstrap replicates among 70 PLCPs subfamily proteins.

The *P. trichocarpa* genomic database was searched and a 1071bp mRNA sequence (gene symbol: LOC7478816) was cloned and named as PtCP5. This gene was annotated as a cathepsin B-like 3 protein using NCBI Reference Sequences (RefSeq). The DNA sequence of PtCP5 was located in chromosome 2 NC_037286.1 (14386340..14389845), including 11 exons. The PtCP5 protein encoded 356 amino acids with the molecular weight 39.53 kD, including a signal peptide of 26 amino acids, MASPLYHGTLFLLVAALFTFHSQVIA. Analysis of the amino acid sequence alignment indicated that the identified amino acid sequence of PtCP5 showed a high degree of homology with several known papain-like cysteine proteases (PLCPs) of cathepsin B-like (CTB3-like) from other plant species and that there is a lack of the corresponding floral structural elements ERFNIN or ERFNAQ in the signal peptide (Figure 1). The conserved catalytic triad of PtCP5, Cys-His-Asn (indicating that all these proteins are functional Cys proteases), was found at similar positions to those of other PLCPs. A phylogenetic tree integrating PLCP subfamily proteins from *A. thaliana* and *P. trichocarpa* was constructed (Figure 2). Phylogenetic tree analysis showed that PtCP5 belonged to CTB 3-like subclasses in the papain-like cystine protein (C1A).

The 1372 bp 5′-flanking promoter region of *PtCP5* gene was cloned from *P.* *trichocarpa* genomic DNA. A predicted analysis of the promoter using Plant CARE and PLACE showed there were several plant hormone-related elements, such as AuxRR-core elements (involved in auxin responsiveness), TATC-box elements (involved in gibberellin responsiveness), ABRE elements (involved in abscisic acid responsiveness) and TGACG-motif and CGTCA-motif elements (involved in methyl jasmonate responsiveness), in the promoter of the *PtCP5* gene, suggesting that PtCP5 may be involved in a variety of regulatory pathways of stress signal responses and plays a key role in regulating the development and stress of *P. tomentosa* (Table 1).

### 2.2. Expression Pattern of the PtCP5 Gene

**Figure 3:**
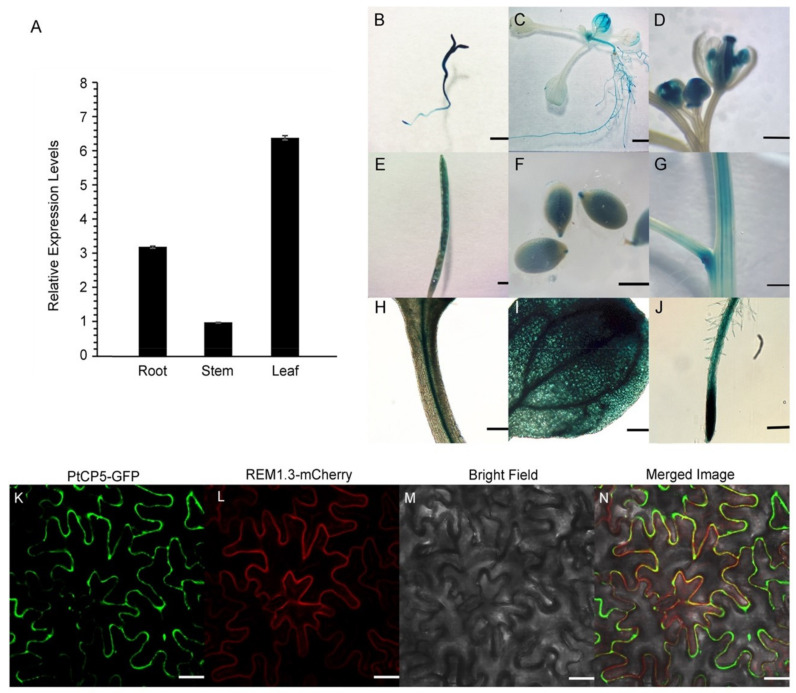
Expression patterns of PtCP5: (**A**) PtCP5 in *P. trichocarpa*. The expression levels were normalized to that of β-actin-F and β-actin-R. Data are mean ± SE (*n* = 3 experiments). (**B**–**J**) *pro*PtCP5:GUS expression patterns in *Arabidopsis*: seedling (**B**); cotyledons (**C**); flower (**D**); siliques (**E**); seed (**F**); stem (**G**); petiole (**H**); leaf (**I**); and root (**J**). (**B**–**G**) bar = 1 mm, (**H**–**J**) bar = 250 μm. (**K–N**) Subcellular fluorescence localization of PtCP5-GFP, bar = 30 μm.

The expression of PtCP5 was detected in the root, stem and leaves of *P. trichocarpa* by qPCR. The results showed a higher expression of PtCP5 in leaves than in the root and stem (Figure 3A). A pBI121-based construct, pBI-ProPtCP5-GUS, was used for further analysis (Figure 3B–J). The expression of GUS was detected in cotyledon, hypocotyl, leaves, anther, pistil, stem vascular tissue, Longhorn fruit, seeds and roots, but not in petals and sepals. There was an especially high expression of GUS in root tips and the embryo and endosperm of seeds.

To determine the subcellular localization of PtCP5, we generated PtCP5–GFP and REM 3.1–mCherry expression vectors, the latter of which is an established marker protein for plasma membrane [24]. And, the two vectors were simultaneously transformed into tobacco leaves by Agrobacterium tumefaciens. The PtCP5-GFP fusion protein was located in the plasma membrane (Figure 3K–N).

These results suggest that the expression of PtCP5 is similar to that of *Arabidopsis* CTB3-like genes and may be widely involved in all stages of plant growth and development.

### 2.3. Overexpressing PtCP5 Obviously Delayed Seed Germination Time and Decreased the Growth Rate in Arabidopsis

**Figure 4:**
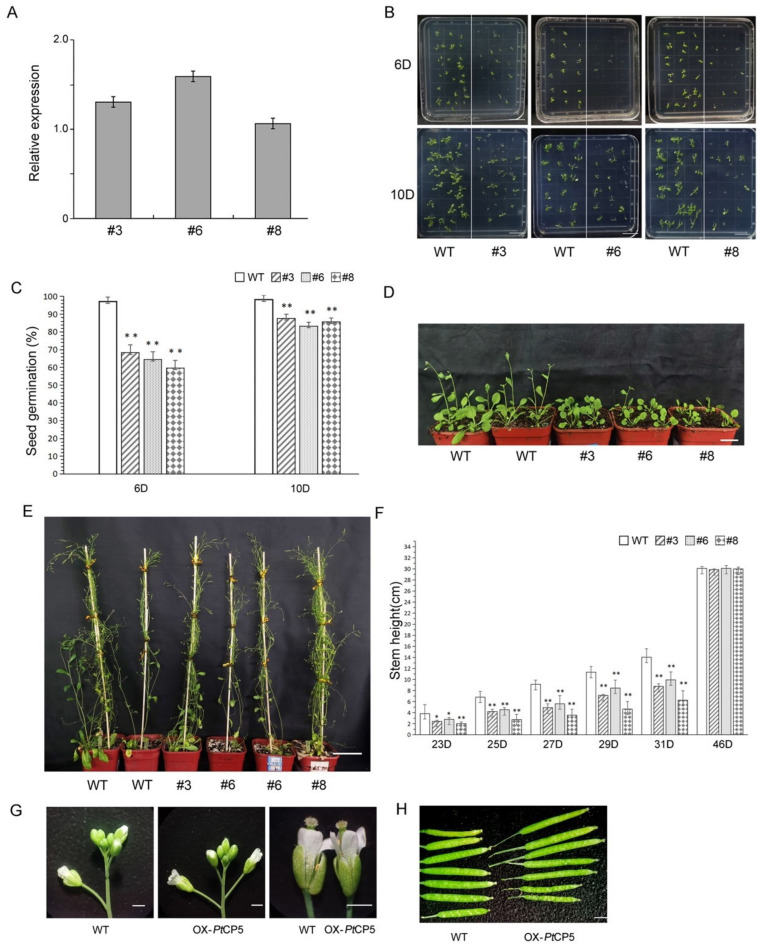
Phenotype of OX-PtCP5 *Arabidopsis*: (**A**) relative expression of *PtCP5* gene in OX-PtCP5. (**B**) Seed germination, bar = 1 cm. (**C**) Seed germination rate. Each medium plate contained about 30 seeds of OX-PtCP5 and wild-type respectively. Error line represents standard deviation (SD), * represents significant difference between OX-PTCP5 and wild-type (*p* < 0.05), ** represents extremely significant difference between OX-PTCP5 and wild-type (*p* < 0.01), similarly hereafter. (**D**) Seedlings, bar = 2 cm. (**E**) Plants, bar = 5 cm. (**F**) Stem height. The number of samples from each strain was about 30. (**G**) Flower, bar = 1 cm. (**H**) Siliques, bar = 1 cm.

Due to the high expression of PtCP5 in seeds, we speculated that it may play an important role in seed germination. A pBI121-based construct, pBI-35S-PtCP5, was used to retrieve overexpressed PtCP5 transgenic *Arabidopsis* (OX-PtCP5). The expression of PtCP5 in independent transgenic plants was detected by qRT–PCR. The transgenic strains (OX-PtCP5: #3, #6, #8) with the highest PtCP5 expression levels were selected for further research (Figure 4A).

The growth phenotype of overexpressing *Arabidopsis* was observed during different developmental stages. The germination time for OX-PtCP5 was obviously delayed. In the case of OX-PtCP5 *Arabidopsis*, the seeds began to germinate on the 6th day, by which point the seeds of the wild-type had completely germinated (Figure 4B,C). About 15% of transgenic seeds could not germinate until the 10th day. Subsequently, the growth rate of OX-PtCP5 was also significantly slower than that of wild-type plants (Figure 4D). The bolting of the wild-type occurred on day 19, when OX-PtCP5 had not yet bolted. The bolting of OX-PtCP5 occurred on day 21. At all stages of plant growth, the stem height of OX-PtCP5 transgenic lines was significantly lower than that of wild-type lines; however, during the final stage of growth, there was no significant difference between the plant height of OX-PtCP5 and wild-type plants (Figure 4E,F). Compared with the wild-type, the OX-PtCP5 transgenic plants showed no significant differences in flowering time, Longhorn fruit bearing and ripening time. Furthermore, there were no significant differences in the morphology of the flowers and Longhorn fruit of OX-PtCP5 transgenic plants when compared with wild-type plants (Figure 4G,H).

**Figure 5:**
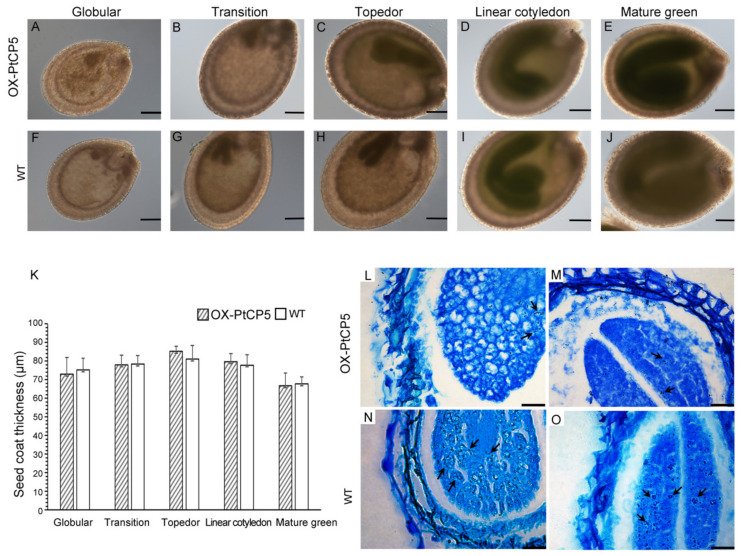
Seeds of OX-PtCP5: (**A–J**) thin sections from dry seeds of OX-PtCP5 and wild-type, bar = 100 μm. (**K**) Seed coat thickness. (**L–O**) PSVs in the cells of cotyledons and hypocotyls, bar = 8 μm.

To analyze whether the reduced germination rate was due to seed damage, the development of OX-PtCP5 and wild-type seeds was analyzed using a differential interference microscope (DIC) (Figure 5). The results showed that the development of OX-PtCP5 seeds (globular, transition, topedor, linear cotyledon and mature green) showed no obvious changes and that the thickness of the seed coats had not changed significantly (Figure 5A–K).

Previous reports suggest that protease may be related to the accumulation and mobilization of seed storage proteins [6,11,12,25,26]. Storage proteins are deposited into protein storage vacuoles (PSVs) during plant seed maturation [27,28,29]. During germination, the storage proteins are rapidly degraded to provide nutrients for use by the embryo. We examined the morphology of protein storage vacuoles (PSV) during the germination of OX-PtCP5 seeds. The number of PSVs in cotyledons and hypocotyls of OX-PtCP5 was evidently lower than in the wild-type, suggesting the accumulation of storage proteins is abnormal in OX-PtCP5 seeds (Figure 5L–O).

These results indicate that the overexpression of PtCP5 also causes significantly accelerated protein mobilization during seed germination, and that decreased PSVs leads to insufficient protein nutrients required for later seed development, which makes the germination of OX-PtCP5 seeds significantly slower than in the wild-type.

### 2.4. Overexpressing PtCP5 Markedly Impaired Pollen Development

**Figure 6:**
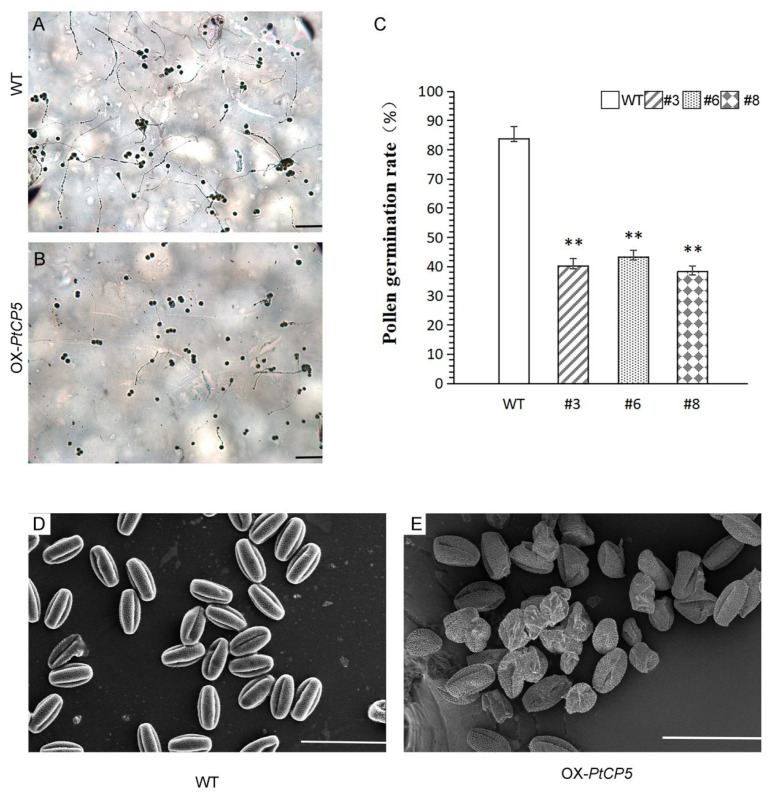
Pollen of OX-PtCP5: (**A**,**B**) pollen germination of OX-PtCP5 and wild-type, bar = 500 μm. (**C**) Pollen germination rate. Pollen samples number of OX-PTCP5 and wild-type were 200 each. ** Represents extremely significant difference between OX-PTCP5 and wild-type (*p* < 0.01). (**D**,**E**) Pollen of wild-type and OX-PtCP5 *Arabidopsis* by scanning electron microscope, bar = 100 μm.

To assess whether the low and slow germination rate of OX-PtCP5 is caused by abnormal pollen development, both the anther structure and the germination rate were analyzed (Figure 6). The germination rate of OX-PtCP5 transgenic pollen was largely decreased when compared to wild-type pollen (Figure 6A–C). Wild-type mature pollen grains were uniformly spheroid and had finely reticulate ornamentation on their surface, but there were a large number of abnormal pollen grains in OX-PtCP5, and the surviving pollen grains exhibited collapsed and gemmate–baculate sculpture without regular reticulate ornamentation (Figure 6D–E). These results show that the overexpression of PtCP5 markedly impairs pollen development, and that the proper expression of PtCP5 is necessary for pollen maturation.

## 3. Discussion

### 3.1. PtCP5 Is Involved in Storage Proteins Accumulated in Seeds

The nutrients provided by plant seeds are the basis for the growth and development of offspring. During seed germination, seed storage proteins are mobilized or degraded as a source of nitrogen for seedling growth. These processes are mainly triggered by cysteine proteinases. In germinating maize and wheat, cysteine proteinases account for up to 90% of the total proteolytic activity of prolamins [1].

Here, we report that a *P.* *trichocarpa* cathepsin B-like cysteine protease PtCP5 is expressed to a high degree in seeds and that the overexpression of PtCP5 decreased seed germination rates in *Arabidopsis*. This is consistent with previous research that shows that the seed-type βVPE is the most important for the maturation of storage proteins in *Arabidopsis* seeds [8]. In the βvpe mutant, the content of mature storage proteins was decreased and storage protein precursors were accumulated. This suggests that PtCP5 participates in the maturation of storage proteins in seeds. Another vacuolar processing enzyme, δVPE, was expressed both specifically and transiently in two cell layers of the seed coat (ii2 and ii3) in the early stages of seed development. δVPE deficiency delayed the targeted cell death but this did not affect seed dormancy or germination [9]. In this study, we found that a PtCP5 promoter can drive Gus expression in leaves, mesophyll cells, roots, stems and seeds. It has been reported that cysteine proteases are involved in the cell death process of specific cell layers during seed coat development in the early stages of seed development [9]. However, there was no significant difference in seed coat thickness between the wild-type (WT) and the overexpressed PtCP5 seeds, suggesting that PtCP5 does not play a key role in seed coat development.

In our results, the seed coat was not affected, but the content of PSV was decreased in overexpressed PtCP5 seeds. These results suggest PtCP5 is responsible for mobilizing stored proteins. It has been reported previously that two cysteine endopeptidases, HvEPA in triticale and HvPap-1 in barley, are responsible for mobilizing stored proteins during seed germination [3,12]. HvPap-1 was localized to the protein bodies and vesicles in the embryo, and it has been shown that it can degrade barley endosperm proteins (hordeins, albumins and globulins). However, unlike in our study, the overexpression of HvPap-1 was shown to decrease the amount of starch in seeds and increase the germination rate, while the silencing of HvPap-1 has displayed an opposite phenotype [13]. This shows that there may be divergent functions between different PLCP proteins.

### 3.2. PtCP5 Is Involved in Pollen Maturation

The formation of fertile pollen in anther locules depends on nutritive contributions from the surrounding sporophytic tissues, and the timely mobilization of proteins also plays an important role in pollen maturation [30,31]. In anthers, many proteases are involved in this process and together they determine pollen maturation [32,33]. Our results show PtCP5 is expressed to a high degree in anther, and that the overexpressing PTCP5 *Arabidopsis* pollen germination rate decreases significantly. The pollen exine, the outer sculptured part of the pollen wall, not only provides a protective barrier against pathogen attack, dehydration and UV irradiation, but it also facilitates pollen recognition and adhesion to the stigma. Our results showed that the overexpressing PTCP5 *Arabidopsis* pollen exine was damaged and that pollen maturation and development was seriously impaired in overexpressing PtCP5 pollen as a result of this. This suggests that PtCP5 is involved in pollen maturation and has an influence on pollen germination.

In this study, subcellular localization analysis showed that PtCP5 was located in the plasma membrane. Through the study of the protein mobilization of PtCP5 transgenic seeds, it was proved that PtCP5 participated in protein mobilization and played a role in protein degradation. In the early stages of seed germination, protein mobilization was too early, which led to the lack of protein nutrients required for late seed development.

## 4. Materials and Methods

### 4.1. Plant Materials and Growth Conditions

The *P. trichocarpa* used in this study is planted in the campus of Beijing Forestry University. *Arabidopsis* seeds were sterilized and were grown on half-strength Murashige and Skoog (1/2 MS) plates with 3% sucrose and 0.6% agar. They were then stratified for 2 days at 4 °C before being transferred to the culture room at 22 °C under a 16/8 light/dark cycle. After germination, 10 d old *Arabidopsis* seedlings were transplanted and grown at a density of four plants per 7 × 7 × 6.5 cm pot containing a mixture of soil and vermiculite (2:1) at 22 °C under a 16/8 light/dark cycle (150 μmol m^−2^s^−1^) and 70% relative humidity.

### 4.2. Cloning of PtCP5 Gene

The suspected cysteine protease gene members were obtained by searching and screening methods according to functional domain division in the *populus tomentosa* genome database website (https://genome.jgi.doe.gov/portal/, accessed on 13 October 2021). Total RNA was extracted from the leaves of *P. trichocarpa,* according to the instructions of the plant total RNA extraction kit (Aidlab, Beijing, China). First-strand cDNA synthesis was performed using M-MLV Reverse Transcriptase and an oligo (dT) primer (Promega, Madison, WI, USA). The PtCP5 cDNA sequence was amplified by PCR using the primers PtCP5-F and PtCP5-R.

### 4.3. Promoter Region Cloning of the PtCP5 Gene

The cloning primer ProPtCP5-F and ProPtCP5-R was designed to amplify the PtCP5 promoter region. Using the genomic DNA of *P. trichocarpa* as a template, the promoter region was cloned with a Genome Walking Kit (Takara, Tokyo, Japan). PCR reactions were conducted according to the manufacturer’s instructions, and products were cloned into the pGEM T- Easy vector (Promega, WI, USA) and sequenced.

### 4.4. Multiple Alignments and Bioinformatic Analyses

The protein sequences of the papain-like cysteine proteases (PLCPs) were obtained from the NCBI protein database (accession numbers are listed in Supplementary Table S1). The isoelectric point (pI) and molecular weight were estimated using the Compute pI/Mw tool from ExPASy (http://web.expasy.org/compute_pi, accessed on 13 October 2021). Proteins signal peptides were predicted using the SignalP4.1 server (http://www.cbs.dtu.dk/services/SignalP/, accessed on 13 October 2021). Comparative and bioinformatic analyses of PtCP5 were carried out online at NCBI. The nucleotide sequence, deduced amino acid sequence and ORF encoded by PtCP5 were each analyzed and a sequence comparison was conducted through database searches using the BLAST. The phylogenetic analysis of PtCP5 and cysteine protease from other species was aligned with CLUSTAL W (1.82) using default parameters. A phylogenetic tree was constructed using MEGA version 7 from CLUSTAL W alignments. The neighbor-joining method was used to construct the tree. Promoter regions of PtCP5 were analyzed for cis-acting regulatory DNA elements using PLACE (http://www.dna.affrc.go.jp/htdocs/PLACE/, accessed on 13 October 2021) and Plant CARE (http://bioinformatics.psb.ugent.be/webtools/plantcare/html/, accessed on 13 October 2021).

### 4.5. Expression Pattern Analysis of PtCP5

To analyze the expression levels of PtCP5, RNA was extracted from the leaf, stem and root of the tissue culture of a *P. trichoderma* plant aged three months. The RT-qPCR analyses were performed using SYBR Green qPCR Mix (TIANGEN, Beijing, China) on an iQ5 Multicolor Real-Time PCR detection system (Bio-Rad Laboratories, Hercules, CA, USA). The PCR conditions were 94 °C for 3 min, 40 cycles at 94 °C for 10 s, 55 °C for 20 s, 72 °C for 20 s, 60 °C for 30 s and 72 °C for 1 min. Data were analyzed using iQ5 (Bio-Rad) software, and differences in gene expression were calculated using the 2^−ΔΔCt^ method. The β-actin, acting as an internal control, was used to quantify the relative expression levels of genes in samples. There were three technical replicates and three biological replicates.

### 4.6. Localization Analysis of PtCP5

For promoter expression analysis, the proPtCP5:GUS construct, including a 1372pb fragment upstream from the initiation codon extracted from *P. trichocarpa* genomic DNA, was cloned into the pBI121 vector and transformed into *Arabidopsis* Col-0. For subcellular localization of PtCP5 in plant cells, GFP and mCherry fusion proteins were observed using a confocal laser scanning microscope (FV1200 Confocal/FLIM/FCS; Leica, Wetzlar, Germany).

### 4.7. Histochemical Staining Analysis

Seedlings were incubated in 90% acetone for 40 min on ice and washed twice with 100 mM sodium phosphate buffer (pH 7). The tissues were then incubated overnight at 37 °C in darkness in a GUS staining buffer containing 100 mM Na_2_HPO_4_ buffer, 1 mM K3(Fe[CN]6), 1 mM K2(Fe[CN]6), 10 mM EDTA, 1%(*v*/*v*) Triton X-100 and 0.5 mg mL^−1^ 5-bromo-4-chloro-3-indolyl-β-D-glucuronic acid. This was followed by cleaning with 75% ethanol.

### 4.8. Plasmid Construction and Plant Transformation

To obtain 35S:PtCP5 transgenic *Arabidopsis* (OX-PtCP5), the cDNA of PtCP5 was cloned into the pBI121 binary vector under the control of a cauliflower mosaic virus (CaMV) 35S promoter and transformed into *Arabidopsis* Col-0 by the floral dip method using Agrobacterium tumefaciens GV3101. The transgenic lines were identified using half-strength MS plates containing 50 mg L^−1^ kanamycin. After selection by kanamycin resistance, the putative transgenic *Arabidopsis* lines were subjected to PCR and RT-PCR assays. A primer pair from binary vector pBI121 near the opposite ends of PtCP5 cDNA, *OX*-PtCP5-F and *OX*-PtCP5-R, was designed and used for the amplification of the inserted fragment of the PtCP5 cDNA. The primer pairs PtCP5-F2 and PtCP5-R2 and β-actin-F2 and β-actin-R2, were used for the analysis of PtCP5 gene expression by RT-PCR. The fragments from PCR and RT-PCR were further confirmed by DNA sequencing. T3 generation homozygous plants were used for subsequent studies.

### 4.9. Physiological Experiments

Three independent batches of seeds were used to confirm the germination rate. Twenty seeds from each batch, arranged in a line, were used for germination comparisons between OX-PtCP5 and wild-type plants. Seeds were separated and sown on the plates based on differences in their germination time. Plant height was measured every 3 days during the bolting period to calculate the average stem height.

### 4.10. Light Microscopic Analysis

Dry seeds from OX-PtCP5 and wild-type plants were vacuum-infiltrated for 1 h with a fixative that consisted of 30% formaldehyde, acetic acid and 50% ethanol. The tissues were then dehydrated in an alcohol gradient series (1 h each at 70, 85, 90 and 100% alcohol) and cleared in a xylene/alcohol gradient series (1 h each at 70, 85, 90 and 100% xylene). The samples were incubated in xylene/paraffin (1:1) overnight at 38 °C and dipped in 58 °C paraffin three times (1 h per incubation). Paraffin-embedded samples were sectioned, stained with toluidine blue and inspected with a DM2500 light microscope.

## 5. Conclusions

In this study, a cathepsin B-like cysteine protease, PtCP5, from *Populus trichocarpa* was expressed in multiple tissues. Ectopic expression analysis of PtCP5 showed decreased storage protein accumulation, delayed seed and pollen germination and delayed plant growth and development in *transgenic Arabidopsis* (OX-PtCP5). In conclusion, functional analysis of PtCP5 in Arabidopsis suggests that PtCP5 participates in plant development by regulating protein mobilization and protein degradation.

## Figures and Tables

**Table 1:** Prediction of major stress-related cis-elements in *PtCP5* promoter.

Element	Core Sequence	Function	Number
TATC-box	TATCCCA	Respond to GA	1
ABRE	ACGTG	Respond to ABA	1
TGACG-motif	TGACG	Respond to methyl jasmonate	1
GC-motif	CCCCCG	enhancer-like element involved in anoxic specific inducibility	1
G-box	TACGTG	cis-acting regulatory element involved in light responsiveness	1
Gap-box	CAAATGAA(A/G)A	part of a light responsive element	1
chs-CMA1a	TTACTTAA	part of a light responsive element	1
AuxRR-core	GGTCCAT	Respond to auxin	1
CGTCA-motif	CGTCA	Respond to methyl jasmonate	1
circadian	CAAAGATATC	Regulation to circadian rhythm	1
MRE	AACCTAA	MYB binding site involved in light responsiveness	2
MYB	CAACCA/CAACAG	Regulation to drought and ABA	2
MYC	CAATTG/CATTTG	Regulation to drought, ABA and cold	2

## Data Availability

The data presented in this study are available on request from the corresponding author.

## Supplementary Materials

The following are available online at https://www.mdpi.com/article/10.3390/ijms222312637/s1.
