# Supplementary material for: How Cysteine Protease Gene PtCP5 Affects Seed Germination by Mobilizing Storage Proteins in Populus trichocarpa
Source: Int J Mol Sci. 2021 Nov 23;22(23):12637. doi: 10.3390/ijms222312637 (PMC8657902; doi:10.3390/ijms222312637)
Supplement: Supplementary file 1 [file ijms-22-12637-s001.zip › ijms-1439667-supplementary.pdf]

Table S1. Sequences and Accession Numbers of Representative Papain-like Cysteine Proteases (PLCPs).

Name	Species	Sequence	Number
CTB1	*Arabidopsis thaliana*	MADSCCIRLHLLASVFLLLFSSFNLQGIAAENLSKQKLTSLILQNEIVKEVNENPNAGWKAAFNDRFANATVAEFKRLLGVIQTPKTAYLGVPIVRHDLSLKLPKEFDARTAWSHCTSIRRILVGYILNNVLLWSTITLWFWFLLGHCGSCWAFGAVESLSDRFCIKYNLNVSLSANDVIACCGLLCGFGCNGGFPMGAWLYFKYHGVVTQECDPYFDNTGCSHPGCEPTYPTPKCERKCVSRNQLWGESKHYGVGAYRINPDPQDIMAEVYKNGPVEVAFTVYEDFAHYKSGVYKYITGTKIGGHAVKLIGWGTSDDGEDYWLLANQWNRSWGDDGYFKIRRGTNECGIEQSVVAGLPSEKNVFKGITTSDDLLVSSV*	NP_563647
CTB2	*Arabidopsis thaliana*	MADNCIRLLHSASVFFCLGLLISSFNLLQGIAAENLSKQKLTSWILQNEIVKEVNENPNAGWKASFNDRFANATVAEFKRLLGVKPTPKTEFLGVPIVSHDISLKLPKEFDARTAWSQCTSIGRILDQGHCGSCWAFGAVESLSDRFCIKYNMNVSLSVNDLLACCGFLCGQGCNGGYPIAAWRYFKHHGVVTEECDPYFDNTGCSHPGCEPAYPTPKCARKCVSGNQLWRESKHYGVSAYKVRSHPDDIMAEVYKNGPVEVAFTVYEDFAHYKSGVYKHITGTNIGGHAVKLIGWGTSDDGEDYWLLANQWNRSWGDDGYFKIRRGTNECGIEHGVVAGLPSDRNVVKGITTSDDLLVSSF*	XP_002889395
CTB3	*Arabidopsis thaliana*	MAVYNTKLCLASVFLLLGLLLAFDLKGIEAESLTKQKLDSKILQDEIVKKVNENPNAGWKAAINDRFSNATVAEFKRLLGVKPTPKKHFLGVPIVSHDPSLKLPKAFDARTAWPQCTSIGNILDQGHCGSCWAFGAVESLSDRFCIQFGMNISLSVNDLLACCGFRCGDGCDGGYPIAAWQYFSYSGVVTEECDPYFDNTGCSHPGCEPAYPTPKCSRKCVSDNKLWSESKHYSVSTYTVKSNPQDIMAEVYKNGPVEVSFTVYEDFAHYKSGVYKHITGSNIGGHAVKLIGWGTSSEGEDYWLMANQWNRGWGDDGYFMIRRGTNECGIEDEPVAGLPSSKNVFRVDTGSNDLPVASV*	XP_023637179.1

CEP1	*Arabidopsis thaliana*	MKRFIVLALCMLMVLETTKGLDFHNKDVESENSLWELYERWRSHHTVARSLEEKAKRFNVFKHNVKHIHETNKKDKSYKLKLNKFGDMTSEEFRRTYAGSNIKHHRMFQGEKKATKSFMYANVNTLPTSVDWRKNGAVTPVKNQGQCGSCWAFSTVVAVEGINQIRTKKLTSLSEQELVDCDTNQNQGCNGGLMDLAFEFIKEKGGLTSELVYPYKASDETCDTNKENAPVVSIDGHEDVPKNSEDDLMKAVANQPVSVAIDAGGSDFQFYSEGVFTGRCGTELNHGVAVVGYGTTIDGTKYWIVKNSWGEEWGEKGYIRMQRGIRHKEGLCGIAMEASYPLKNSNTNPSRLSLDSLKDEL*	XP_002864049
XCP2	*Arabidopsis thaliana*	MALSSPSRILCFALALSAASLSLSFASSHDYSIVGYSPEDLESHDKLIELFENWISNFEKAYETVEEKFLRFEVFKDNLKHIDETNKKGKSYWLGLNEFADLSHEEFKKMYLGLKTDIVRRDEERSYAEFAYRDVEAVPKSVDWRKKGAVAEVKNQGSCGSCWAFSTVAAVEGINKIVTGNLTTLSEQELIDCDTTYNNGCNGGLMDYAFEYIVKNGGLRKEEDYPYSMEEGTCEMQKDESETVTINGHQDVPTNDEKSLLKALAHQPLSVAIDASGREFQFYSGGVFDGRCGVDLDHGVAAVGYGSSKGSDYIIVKNSWGPKWGEKGYIRLKRNTGKPEGLCGINKMASFPTKTK*	NP_564126
XBCP3	*Arabidopsis thaliana*	MSMSSSSFISLTFFFLLLVSSSSSSDDISELFDDWCQKHGKTYGSEEERQQRIQIFKDNHDFVTQHNLITNATYSLSLNAFADLTHHEFKASRLGLSVSAPSVIMASKGQSLGGSVKVPDSVDWRKKGAVTNVKDQGSCGACWSFSATGAMEGINQIVTGDLISLSEQELIDCDKSYNAGCNGGLMDYAFEFVIKNHGIDTEKDYPYQERDGTCKKDKLKQKVVTIDSYAGVKSNDEKALMEAVAAQPVSVGICGSERAFQLYSRGIFSGPCSTSLDHAVLIVGYGSQNGVDYWIVKNSWGKSWGMDGFMHMQRNTENSDGVCGINMLASYPIKTHPNPPPPSPPGPTKCNLFTYCSSGETCCCARELFGLCFSWKCCEIESAVCCKDGRHCCPHDYPVCDTTRSLCLKKTGNFTAIKPFWKKNSSKQLGRFEEWVM*	AAK71314
RD21A	*Arabidopsis thaliana*	MGFLKPTMAILFLAMVAVSSAVDMSIISYDEKHGVSTTGGRSEAEVMSIYEAWLVKHGKAQSQNSLVEKDRRFEIFKDNLRFVDEHNEKNLSYRLGLTRFADLTNDEYRSKYLGAKMEKKGERRTSLRYEARVGDELPESIDWRKKGAVAEVKDQGGCGSCWAFSTIGAVEGINQIVTGDLITLSEQELVDCDTSYNEGCNGGLMDYAFEFIIKNGGIDTDKDYPYKGVDGTCDQIRKNAKVVTIDSYEDVPTYSEESLKKAVAHQPISIAIEAGGRAFQLYDSGIFDGSCGTQLDHGVVAVGYGTENGKDYWIVRNSWGKSWGESGYLRMARNIASSSGKCGIAIEPSYPIKNGENPPNPGPSPPSPIKPPTQCDSYYTCPESNTCCCLFEYGKYCFAWGCCPLEAATCCDDNYSCCPHEY	XP_020866872

		PVCDLDQGTCLLSKNSPFSVKALKRKPATPFWSQGRKNIA*	
THI1	*Arabidops*<br>*is thaliana*	MLNVLRNSNLTLAVLICFVLIASKLCSVDSSVYDPHKTLKQRFEKWLK<br>THSKLYGGRDEWMLRFGIYQSNVQLIDYINSLHLPFKLTDNRFADMT<br>NSEFKAHFLGLNTSSLRLHKKQRPVCDPAGNVPDAVDWRTQGAVTP<br>IRNQGKCGGCWAFSAVAAIEGINKIKTGNLVSLSEQQLIDCDVGTYNK<br>GCSGGLMETAFEFIKTNGGLATETDYPYTGIEGTCDQEKSKNKVVTI<br>QGYQKVAQNEASLQIAAAQQPVSVGIDAGGFIFQLYSSGVFTNYCGT<br>NLNHGVTVVGYGVEGDQKYWIVKNSWGTGWGEEGYIRMERGVSE<br>DTGKCGIAMMASYPLQ*	NP_563764
SAG12	*Arabidops*<br>*is thaliana*	MALKHMQIFLFVAIFSSFCFSITLSRPLDNELIMQKRHIEWMTKHGRV<br>YADVKEENNRYVVFKNNVERIEHLNSIPAGRTFKLAVNQFADLTNDEF<br>CSMYTGFKGVSALSSQSQTKMSPFRYQNVSSGALPVSVDWRKKGA<br>VTPIKNQGSCGCCWAFSAVAAIEGATQIKKGKLISLSEQQLVDCDTND<br>FGCEGGLMDTAFEHIKATGGLTTESDYPYKGEDATCNSKKTNPKATS<br>ITGYEDVPVNDEQALMKAVAHQPVSVGIEGGGFDFQFYSSGVFTGE<br>CTTYLDHAVTAIGYGESTNGSKYWIIKNSWGTKWGESGYMRIQKDV<br>KDKQGLCGLAMKASYPTI*	AAC49135
RD19A	*Arabidops*<br>*is thaliana*	MDRLKLYFSVFVLSFFIVSVSSSDVNDGDDLVIRQVVGGAEPQVLTSE<br>DHFSLFKRKFGKVYASNEEHDYRFSVFKANLRRARRHQKLDPSATH<br>GVTQFSDLTRSEFRKKHLGVRSGFKLPKDANKAPILPTENLPEDFDW<br>RDHGAVTPVKNQGSCGSCWSFSATGALEGANFLATGKLVSLSEQQL<br>VDCDHECDPEEADSCDSGCNGGLMNSAFEYTLKTGGLMKEEDYPY<br>TGKDGKTCKLDKSKIVASVSNFSVISIDEEQIAANLVKNGPLAVAINAG<br>YMQTYIGGVSCPYICTRRLNHGVLLVGYGAAGYAPARFKEKPYWIIK<br>NSWGETWGENGFYKICKGRNICGVDSMVSTVAATVSTTAH*	AAM65162

AALP	*Arabidopsis thaliana*	MSAKTILSSVVLVVLVAASAAANIGFDESNPIRMVSDGLREVEESVSQILGQSRHVLSFARFTHRYGKKYQNVEEMKLRFSIFKENLDLIRSTNKKGLSYKLGVNQFADLTWQEFQRTKLGAAQNCSATLKGSHKVTEAALPETKDWREDGIVSPVKDQGGCGSCWTFSTTGALEAAYHQAFGKGISLSEQQLVDCAGAFNNYGCNGGLPSQAFEYIKSNGGLDTEKAYPYTGKDETCKFSAENVGVQVLNSVNITLGAEDELKHAVGLVRPVSIAFEVIHSFRLYKSGVYTDSHCGSTPMDVNHAVLAVGYGVEDGVPYWLIKNSWGADWGDKGYFKMEMGKNMCGIATCASYPVVA*	NP_568921
9.1	Populus trichocarpa	MLCGQQATAEEPVSKLKLNSRILQDSIVQKVNENPKAGWEATMNPQFSNYSVGEFKYLLGVKQTPRKELRGVPLLRHPKSMKLPIEFDARTAWPHCSTIGRILDQGHCGSCWAFGAVESLSDRFCIHYGMNLSLSVNDLLACCGWMCGAGCDGGSPIDAWRYFVQSGVVTEECDPYFDDIGCSHPGCEPGFPTPKCERKCADKNKLWAESKHFSVNAYRIDSDPHSIMAEVSSNGPVEVAFTVYEDFAHYKSGVYKHITGDAMGGHAVKLIGWGTSEDGEDYWLLANQWNRGWGDDGYFKIKRGTNECGIEGAVVAGLPSTRNLVREVAGIDGHEHATA*	XP_006375410
9.2	Populus trichocarpa	METSLCFSTLLLLLIGAIFTFQSQVIAVEPVSDLKLNSRILQDSILKKVNGNPKAGWKATMNHHFSNYTVAQFKYLLGVKPTPKEELRGIPVISHPKSLRLPEEFDARTAWPQCSTIGKILDQGHCGSCWAFGAVESLSDRFCIHYGMNISLSVNDLLACCGFLCGSGCNGGYPISAWRYFVHHGVVTEECDPYFDDIGCSHPGCEPGYPTPKCARKCVNKNQLWKKSKHYGVKPYRIDSDPESIMAEIYKNGPVEVAFTVYEDFAHYKSGVYKHITGGMMGGHAVKLIGWGTSEDGEAYWLLANQWNRGWGDDGYFKIRRGTNECGIEGDVVAGLPSTRNLVREVVSVDAREDASA*	XP_002301457
PtCP5	Populus trichocarpa	MASPLYHGTLFLLVAALFTFHSQVIAVEPVSNLKLNSRILQDSIVQKVNENPNAGWEATMNPQFSNYSVGEFKYLLGVKPTPKKELRGVPLVRHPKSMKLPKEFDARTAWSHCSTIGRILDQGHCGSCWAFGAVESLSDRFCIHYGMNLSLSVNDLLACCGWMCGDGCDGGYPIDAWRYFVQSGVVTEECDPYFDDIGCSHPGCEPGFPTPKCERKCADKNKLWAESKHFSVNAYRIDSDPHSIMAEVSMNGPVEVAFTVYEDFAHYKSGVYKHITGDVMGGHAVKLIGWGTSDDGEDYWLLANQWNRGWGDDGYFKIRRGTNECGIEEDVVAGLPSTRNLVREIAKIDAHEHASA*	
